# 

**Published:** 1847-07

**Authors:** 


*New York Medical and Surgical Reporter.*—This Journal is now discontinued.

